# Glycine Transporters and Receptors as Targets for Analgesics

**DOI:** 10.3390/biom11111676

**Published:** 2021-11-11

**Authors:** Robert J. Harvey, Robert J. Vandenberg

**Affiliations:** 1 School of Health and Behavioural Sciences, University of the Sunshine Coast, Maroochydore, QLD 4556, Australia; 2 Sunshine Coast Health Institute, Birtinya, QLD 4558, Australia; 3 School of Medical Sciences, Faculty of Medicine and Health, The University of Sydney, Sydney, NSW 2006, Australia; robert.vandenberg@sydney.edu.au

The suitability of modulating glycinergic neurotransmission for the treatment of inflammatory and chronic pain has gained widespread recognition, with glycine receptors (GlyRs) and glycine transporters (GlyT1 and GlyT2) now considered key therapeutic targets [1,2]. This Special Issue explored a range of perspectives on this topic, considering recent advances in understanding the pathophysiology of glycinergic neurotransmission in pain states, the structure and function of transporters and receptors, mechanisms of transporter and receptor regulation, how potential drugs provide analgesia in animal models of pain, and the current state of clinical trials.

The scene is set in a review by Cioffi [3], who discussed several inhibitors of GlyT1 and GlyT2 that have demonstrated broad analgesic efficacy in several preclinical models of acute and chronic pain. Importantly, Cioffi highlighted mechanism-based safety concerns for GlyT2 inhibitors relating to the following: (1) poor oral bioavailability and CNS permeability; (2) the lethality of the GlyT2 knockout mouse strain; and (3) the involvement of mutations in the human GlyT2 gene in a human neuromuscular disorder, startle disease, or hyperekplexia. However, Cioffi also made a compelling case for the development of partial and/or reversible GlyT2 inhibitors, which would avoid the potential harm of complete GlyT2 inhibition. Strong arguments were also made for developing and testing non-selective, bispecific GlyT1/GlyT2 inhibitors, which together would be predicted to produce a synergistic effect, leading to significant increases in synaptic glycine concentrations and augmented inhibitory glycinergic signaling in the spinal cord dorsal horn.

Regarding the pharmacology of GlyTs, Barsch and colleagues [4] explored the pharmacology of propacetamol, a non-opioid analgesic used in post-operative care and to treat mild pain conditions. Propacetamol is hydrolysed in vivo into *N*,*N*-diethylglycine (DEG) and acetaminophen. Since DEG has structural similarities to GlyT1 substrates, Barsch et al. analyzed the effects of propacetamol, acetaminophen, and DEG on GlyR and GlyT function, using a two-electrode voltage-clamp approach in *Xenopus laevis* oocytes. Although propacetamol and acetaminophen did not affect GlyR/GlyT function, DEG acted as a low-affinity substrate for both GlyT1 and GlyT2, with EC_50_ values of 5–8 mM. DEG also acted as a positive allosteric modulator (PAM) of GlyRs containing the α1, but not α2 or α3 subunits at low millimolar concentrations. Taken together, these data suggest that the action(s) of propacetamol metabolites on GlyRs and GlyTs are relevant to the analgesic effects of this drug.

Dual-action modulators of GlyRs and GlyTs were also studied by Sheipouri and colleagues [5] using an inventive new system for assessing these novel therapeutics. By co-expressing α1 subunit GlyR homomers with GlyT1 or GlyT2 in *Xenopus laevis* oocytes, Sheipouri et al. were able to use voltage-clamp recordings to measure the impact of GlyT expression on GlyR function, and the effects of GlyR/GlyT modulators. Curiously, increases in GlyT density in close proximity to GlyRs diminished receptor currents. This effect was found to be glycine- and Na^+^-dependent and was not observed when GlyTs were blocked with selective inhibitors (e.g., ALX-5407 for GlyT1 or ORG-25543 for GlyT2). Importantly, partial inhibition of GlyT2 and modest GlyR α1 potentiation using a dual-action compound (C18-cis-ω9-glycine) was as effective at restoring GlyR currents as a full and potent single-target GlyT2 inhibitor (ORG-25543) or GlyR α1 PAM (C18-cis-ω7-glycine). This study highlights the importance of considering a synaptic context when testing candidate molecules affecting GlyR or GlyT2 function, and provides a novel testing system for assessing the effects of dual-action GlyR/GlyT modulators.

Regarding animal models of pain, Kuo et al. [6] explored the use of an orally active GlyT2 inhibitor (a 3-pyridyl amide derivative of ORG25543) in a rodent model of chemotherapy-induced peripheral neuropathy (CIPN). CIPN is a major dose-limiting side effect of several first-line chemotherapeutic agents, including cisplatin, affecting several million patients annually worldwide. Since there are currently no effective treatments for alleviating CIPN, Kuo et al. compared the efficacy of the GlyT2 inhibitor to representative drugs from three different analgesic/adjuvant drug classes: pregabalin (Ca^2+^ channel inhibitor), duloxetine (serotonin and norepinephrine reuptake inhibitor), and indomethacin (NSAID). The GlyT2 inhibitor out-performed the other drugs, alleviating both mechanical allodynia and hyperalgesia in the bilateral hindpaws at a dose of 10 mg/kg. However, this effect was not present at higher or lower doses, suggesting that further optimisation is required before these findings can be translated into the clinic.

Finally, Zeilhofer and colleagues provided an expert perspective on the utility of modulating inhibitory α3 subunit GlyRs in inflammatory and chronic pain [7]. This GlyR subtype is highly enriched in the superficial layers of the spinal dorsal horn, a key site of nociceptive processing [8]. Zeihofer and colleagues comprehensively reviewed the evidence linking α3 subunit GlyRs to PGE_2_ signalling pathways and central inflammatory pain sensitisation. They also highlighted new important genetic evidence of glycinergic pain control in humans, namely the decreased pain thresholds observed in individuals with startle disease/hyperekplexia with confirmed mutations in either the GlyR α1 or GlyT2 genes [9]. Important perspectives were also given on screening for synthetic GlyR modulators with potential analgesic effects, for example the use of Zn^2+^ chelators to reduce the rate of false positive hits, since GlyR function is modulated by exogenous and endogenous Zn^2+^ in a bidirectional fashion. We would also add to this the importance of screening and testing candidate compounds on native heteromeric GlyR conformations, i.e., α1β versus α3β. Lastly, the authors closed with pertinent arguments concerning the desired selectivity of GlyR PAMs, namely whether GlyR α3 specificity is even required or preferred? There are certainly strong arguments that spinal cord synapses also contain α1β GlyRs, which are not subject to inhibition via EP2 receptor/PGE_2_-stimulated protein kinase A phosphorylation [8]. These α1β GlyRs could, therefore, be selectively targeted to potentiate inhibition in the dorsal horn independent of pathways modulating GlyR α3β.

Taken together, these original research and review articles highlight that GlyRs and GlyTs remain outstanding new targets for modulation by existing and novel classes of analgesics. Since GlyR and GlyT modulators do not target opioid receptor pathways, they offer a realistic pathway towards finding effective, safe, and non-addictive strategies to managing chronic and inflammatory pain. 
